# Blue whales respond to simulated mid-frequency military sonar

**DOI:** 10.1098/rspb.2013.0657

**Published:** 2013-08-22

**Authors:** Jeremy A. Goldbogen, Brandon L. Southall, Stacy L. DeRuiter, John Calambokidis, Ari S. Friedlaender, Elliott L. Hazen, Erin A. Falcone, Gregory S. Schorr, Annie Douglas, David J. Moretti, Chris Kyburg, Megan F. McKenna, Peter L. Tyack

**Affiliations:** 1 Cascadia Research Collective, 218 1/2 W. 4th Avenue, Olympia, WA 98501, USA; 2 Southall Environmental Associates Inc., 9099 Soquel Drive, Suite 8, Aptos, CA 95003, USA; 3 Long Marine Laboratory, University of California, Santa Cruz, Institute of Marine Sciences, 100 Shaffer Road, Santa Cruz, CA 95060, USA; 4 Duke University Marine Laboratory, 135 Duke Marine Laboratory Road, Beaufort, NC 28516, USA; 5 National Oceanic and Atmospheric Administration, Pacific Grove, CA, USA; 6 Division Newport, Naval Undersea Warfare Center, Newport, RI, USA; 7 Spawar Systems Center, Pacific, Code 7175, 53475 Strothe Road, San Diego, CA 92152, USA; 8 National Park Service, 1201 Oakridge Drive, Suite 100, Fort Collins, CO 80525, USA; 9 CREEM, The Observatory, Buchanan Gardens, University of St Andrews, Fife KY16 9LZ, UK; 10 Sea Mammal Research Unit, Scottish Oceans Institute, University of St Andrews, Fife KY16 9LZ, UK

**Keywords:** blue whale, military sonar, underwater noise, sensory ecology, foraging, bio-logging

## Abstract

Mid-frequency military (1–10 kHz) sonars have been associated with lethal mass strandings of deep-diving toothed whales, but the effects on endangered baleen whale species are virtually unknown. Here, we used controlled exposure experiments with simulated military sonar and other mid-frequency sounds to measure behavioural responses of tagged blue whales (*Balaenoptera musculus*) in feeding areas within the Southern California Bight. Despite using source levels orders of magnitude below some operational military systems, our results demonstrate that mid-frequency sound can significantly affect blue whale behaviour, especially during deep feeding modes. When a response occurred, behavioural changes varied widely from cessation of deep feeding to increased swimming speed and directed travel away from the sound source. The variability of these behavioural responses was largely influenced by a complex interaction of behavioural state, the type of mid-frequency sound and received sound level. Sonar-induced disruption of feeding and displacement from high-quality prey patches could have significant and previously undocumented impacts on baleen whale foraging ecology, individual fitness and population health.

## 1. Introduction

Mounting evidence suggests that anthropogenic noise can harm marine life [1–6]. The first concerns were that low-frequency anthropogenic noise could mask calling behaviour in baleen whales (Mysticeti), thereby reducing their communication range [7,8], and that intense levels of noise could also damage hearing [1]. These effects continue to be a high priority for the management and conservation of cetaceans owing to worldwide shipping traffic and resource extraction in environmentally sensitive and critical habitats such as the Arctic [9]. Recent mass stranding events and mortality of cetaceans have been linked to mid-frequency active (MFA) military sonar (i.e. range: 1–10 kHz) [3,10–13]. The strong impact of mid-frequency naval sonar is puzzling because the frequency of the sounds and best hearing of many toothed whales (Odontoceti) are much higher than mid-frequency sonar [14], and the communication band of mysticetes is generally much lower. Most environmental reviews have discounted the effects of noise outside the predominant communication band for many species, especially for baleen whales, because they are rarely represented in sonar-induced stranding events [15]. Given the lack of a comprehensive and mechanistic understanding of how mid-frequency affects different species, empirical measurements of behavioural response to these sounds are critically needed and should be directly determined across taxa [15].

Although most animals involved in mass stranding events associated with mid-frequency sonar are deep-diving beaked whales (Ziphiidae), several cases have included baleen whales [13]. In some stranded whales, there appears to be a common pattern consisting of gas-bubble lesions and fat emboli inside the body [10,16] that are thought to arise from major changes in diving behaviour and physiology [17,18]. The temporal patterns and geographical scales of most stranding events suggest that behavioural response to sound exposure plays a key role in a cascade of events leading to disorientation, injury, stranding and mortality. Previous evaluations of behavioural response have included passive acoustic monitoring to quantify changes in vocal behaviour of groups of animals during mid-frequency sonar exposure [19,20]. These studies provide strong evidence for modified behaviour during sonar exposure, but they do not assess fine-scale changes in individual whales. By using animal-borne tags that simultaneously measure body movement and the proximate acoustic environment at high-resolution, researchers have directly measured behavioural response during sound exposure [21–26]. Although these types of controlled exposure experiments (CEEs) have demonstrated that odontocetes, especially beaked whales, can be sensitive to mid-frequency sounds [21], no CEEs testing responses of baleen whales to mid-frequency sonars have, to our knowledge, yet been performed. Therefore, we conducted CEEs on tagged blue whales in the Southern California Bight to test the hypothesis that low-frequency baleen whales do not respond to mid-frequency sound.

## 2. Material and methods

### (a) Controlled exposure experiment methodology

We assessed the response of blue whales to anthropogenic sound using CEEs. This research paradigm involved: (i) deployment of digital tags on a focal individual, (ii) pre-exposure period to obtain baseline behaviour data (30 min), (iii) exposure period (30 min), and (iv) post-exposure monitoring period (30 min) [21,23]. During summer and autumn 2010, we performed CEEs on tagged blue whales off the coast of Southern California. The research vessel configuration, sound source specifications and CEE methods are described in detail by Southall *et al*. [23]; they are briefly discussed here. We used a sound source deployed from a primary research vessel to project simulated military sonar (MFA sonar) signals and pseudo-random noise (PRN) with similar frequency bands and temporal patterns. As discussed by Southall *et al*. [23], our simulated MFA signals were intended to imitate actual operational sonar used by the United States Navy, but at significantly lower source levels. The digital tags were attached to animals from independently operating rigid-hull inflatable vessels with operations coordinated by, but not centralized on, the command and control vessel. A custom-built, hand-deployable, 15-element vertical line array of active transducers was selected as the source configuration for projecting mid-frequency experimental signals.

Tagged whales were exposed (minimum range of 200 m) to one of two stimuli: simulated MFA or PRN (both within the same approximate frequency band 3.5–4.0 kHz). Either simulated MFA or PRN signals were transmitted at a starting source level of 160 dB @ 1 m, with one transmission onset every 25 s ramped up by 3 dB per transmission to maximum output levels for each signal. We programmed the sound source to generate signals every 25 s during the 30 min CEE, ramping up, in 3 dB increments, from 160 to 210 dB re 1 µPa (r.m.s.). The MFA signal was 1.6 s in total duration, consisting of a 3.5–3.6 kHz linear FM sweep (0.5 s), then a 3.75 kHz tone (0.5 s), a 0.1 s delay and finally a 4.0 kHz tone (0.5 s); it was projected at a maximum source level of 210 dB @ 1 m. The PRN signal was 1.4 s in total duration, consisting of 3.5 to 4.05 Hz band-limited noise (1.0 s), a 0.1 s delay and finally 3.5 to 4.0 Hz band-limited noise (0.3 s); it was projected at a maximum source level of 206 dB @ 1 m. The use of a ramp-up protocol was a permit requirement and is part of several differences (notably including maximum source level and differential movement during transmissions) from some real military sources; subsequent progressions of experimental approaches should include operational source with greater contextual similarities to real operations [27].

### (b) Kinematic, behavioural and environmental context analyses for tagged blue whales

**Figure 1.**
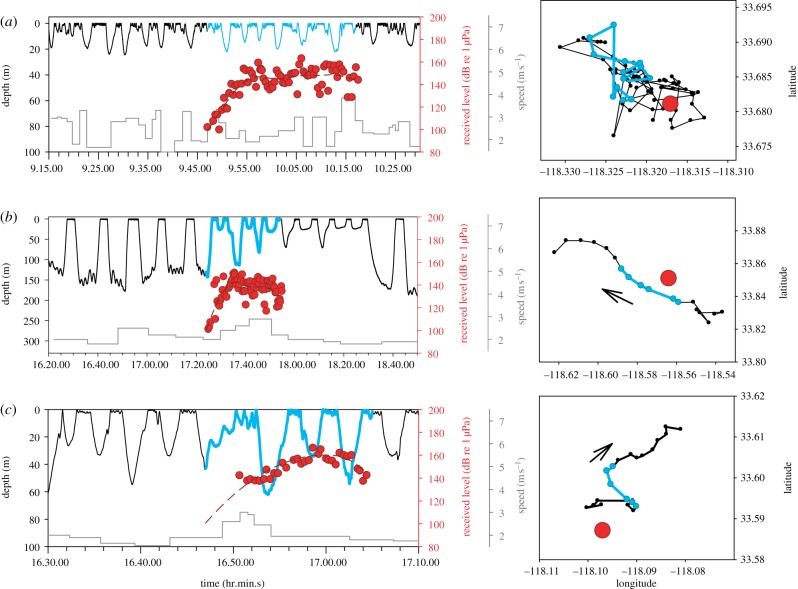
Examples of behavioural dynamics of tagged blue whales during CEEs. (*a*) Simulated mid-frequency sonar during surface feeding, (*b*) PRN during deep feeding, and (*c*) simulated mid-frequency sonar during travel. Dive profiles (left panels, black solid lines), average dive speed (grey lines), received sound levels (each red circle represents a single ping detected by the tag), and the whale's horizontal movement (right panels, each circle represents surface location recording) are shown as a function of time. The sound exposure periods are highlighted in blue on each dive profile and track line. Red dashed lines are spline functions fit though the received sound-level data and extrapolated to include the entire exposure period where appropriate. The location of the sound source at the beginning of playback is highlighted by the large red circle in the right panels. Note that the received sound levels in (*c*) represent only a minimum estimate and the maximum instantaneous swimming speeds exceeded 4 m s^–1^ during the ascent phase of the first exposure dive (see details in the electronic supplementary material).

In order to quantify the fine-scale movement and acoustic environment of focal individuals, we attached multi-sensor digital tags [28,29] containing a suite of sensors that allowed us to estimate body orientation [28], swimming activity, depth, speed [29] and received levels of sound [21,23]. We divided the resulting 54 kinematic, acoustic and environmental variables into three sets: dive behaviour, body orientation and horizontal movement. We used two types of suction-cup attached, multi-sensor digital tags called DTAGs [28] and Bioacoustic Probes [29,30], to study the acoustic environment and movement of blue whales during CEEs. Of the 17 CEEs performed in this study, only one whale was tagged with a Bioacoustic Probe (figure 1*c*). The remaining 16 CEEs involved blue whales tagged with DTAGs. The DTAGs contained a suite of sensors that included stereo hydrophones (sampling frequency, ƒ > 64 kHz), a pressure transducer and tri-axial magnetometers and accelerometers. The non-acoustic auxiliary sensors were sampled at 50 Hz and then decimated to 5 Hz for the analyses below. The Bioacoustic Probe sampled sound pressure at 8 kHz and the auxiliary sensors (dual-axis accelerometers) were sampled at 1 Hz. Owing to the limited sampling frequency of the hydrophone in the Bioacoustic Probe, the received sound levels reported in figure 1*c* represent minimum estimates.

A series of behavioural and environmental parameters were analysed during each blue whale dive following previously published methods [29,31–34]. The data from the suction-cup attached DTAGs were processed and calibrated following the methods of Johnson & Tyack [28]. Body orientation was estimated using the tri-axial accelerometers and magnetometers [28]. Speed was estimated using the flow noise detected by the hydrophone using the necessary calibration procedures for each tag deployment. This involved analysing the speed of the body during steep body pitch angles (vertical velocity divided by the sine of the body pitch angle) and correlating the magnitude of flow noise with the speed vector, a method that has been used to estimate speed in several studies [29,33,35]. Acoustic analyses followed the methodology of Southall *et al*. [23] and Tyack *et al*. [21]. Specifically, we measured received level of sound exposure as the maximum r.m.s. sound pressure level (in dB re 1 µPa) in any one 200 ms time period during the signal duration. Signal duration was defined as the time period during which the signal-to-noise ratio was at least 6 dB. Before level measurements were taken, the signals were filtered with a one third-octave filter spanning the CEE sound frequencies (512-point finite impulse response filter, 3300–4158 Hz).

The specific behavioural and environmental parameters in our analyses included the following: maximum depth, dive duration, descent time, bottom time, ascent time, post-dive surface time, number of lunges per dive, the proportion of the descent spent gliding, average speed during descent, average speed during ascent, average pitch during descent, greatest change in pitch during descent (Δ descent pitch), average pitch during ascent, greatest change in pitch during ascent (Δ ascent pitch), average roll during descent, greatest change in roll during descent (Δ descent roll), average roll during ascent, greatest change in roll during ascent (Δ ascent roll), average heading during descent, greatest change in heading during descent (Δ descent heading), average heading during ascent, greatest change in heading during ascent (Δ descent heading), horizontal dive speed, horizontal speed during surface series, angular trajectory (horizontal turning rate 1) and mean rotation rate (horizontal turning rate 2), number of received pings (from sound exposure), minimum received level, mean received level, maximum received level, depth of the seafloor at the location of the sound source, distance between the sound source and tagged whale at the beginning and end of each dive, photo identification, group type comprising the tagged whale (single, pair, three-way), number and group composition of other cetaceans within 1 km of the tagged whale, and behavioural state of the tagged whale at the moment of initial sound exposure. Behavioural state was determined from the tag data and took the form of one of three broad categories: deep feeding, surface feeding and non-feeding (i.e. travelling or social). The presence of a lunge feeding event was required to categorize the dive as a feeding dive and a maximum dive depth of 50 m was chosen to distinguish between surface feeding and deep feeding behavioural states. Social animals included either paired whales within several body lengths distance from one another or vocalizing whales as indicated from the tag's acoustic record.

### (c) Statistical analyses

We used a combination of principal component analyses (PCAs) and generalized additive mixed models (GAMMs) to assess the effect of sonar playback on 54 categorical and continuous behavioural metrics. PCAs were conducted using ‘princomp’ in the stats package of the open source software R (v. 2.15.1). Behavioural metrics were assessed on a dive-by-dive basis and summarized into three categories prior to PCAs: (i) dive behaviour metrics, (ii) angular (body orientation) metrics, (iii) horizontal behaviour metrics. PCA eigenvectors with greater than 10% of variance explained were used as response variables in controlled exposure GAMMs. We fit two GAMMs per eigenvector, one assessing treatment status as a function of playback period (equation (2.1)—before playback, during playback and after playback) and one quantifying response as a function of playback type (equation (2.2)—during playback with categorical playback type—MFA or PRN).2.1
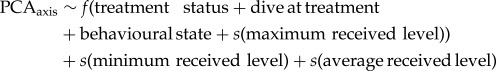
and2.2
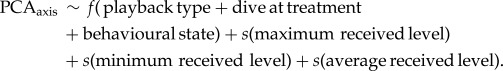
This statistical approach allowed us to assess whether there was a behavioural response if treatment status was significant, whether there was a difference between MFA and PRN if playback type was significant and whether received level influenced behaviour. PCA results are summarized in table 1 and GAMM results are summarized in table 2 (see also the electronic supplementary material, table S1).

**Table 1.** PCA results of behavioural metrics. (Only eigenvectors (EV) that explained more than 10% variance are shown for each parameter group.)

dive metrics	EV1	orientation metrics	EV1	EV2	horizontal metrics	EV1	EV2	EV3
dive time	−0.387	descent pitch	−0.392		horizontal speed (dive)		−0.437	0.523
maximum depth	−0.381	descent roll		0.328	surface speed (surface)		−0.244	0.686
post-dive surface time	−0.346	descent heading	−0.121	−0.521	horizontal turning rate 1		−0.651	−0.218
descent time	−0.340	Δ descent pitch	0.328	−0.230	horizontal turning rate 2		−0.568	−0.452
ascent time	−0.321	Δ descent roll	0.324		distance to sound source (dive start)	0.574		
bottom time	−0.339	Δ descent heading	0.306	0.145	distance to sound source (dive end)	0.574		
lunges	−0.343	ascent pitch	0.397		Δ distance to sound source	0.576		
breaths	−0.366	ascent roll		−0.335				
		ascent heading	−0.102	−0.578				
		Δ ascent pitch	0.351	−0.279				
		Δ ascent roll	0.339	0.12				
		Δ ascent heading	0.341					
proportion of variance	0.758		0.388	0.122		0.428	0.281	0.212

**Table 2.** Summary of significant response metrics from paired PCA-GAMM models. Results of paired PCA-GAMM models examining the effects of sound playback on multiple behavioural metrics. (All statistical results shown in the table are from analysis of the first eigenvector within each response metric grouping (‘*n*’ corresponds to the number of dives analysed across all individuals). Each row represents a tested hypothesis rather than a unique model.)

hypothesis	response metric	PCA-GAMM	PCA variance	*n*	*p*-value	*r*^2^
behaviour changes during sound exposure	dive	before/during/after	0.76	430	0.038	0.14
behaviour changes during sound exposure	orientation	before/during/after	0.39	430	0.019	0.04
behaviour changes during sound exposure	horizontal displacement	before/during/after	0.43	418	<0.005	0.07
effect of sound type (MFA versus PRN)	dive	during	0.76	88	0.033	0.38
effect of behavioural state	dive	before/during/after	0.76	430	0.0374	0.14
effect of behavioural state	dive	during	0.76	88	0.0454	0.38
effect of maximum dive received level	dive	during	0.76	88	<0.005	0.38
effect of minimum dive received level	dive	during	0.76	88	0.0369	0.38

## 3. Results and discussion

**Figure 2.**
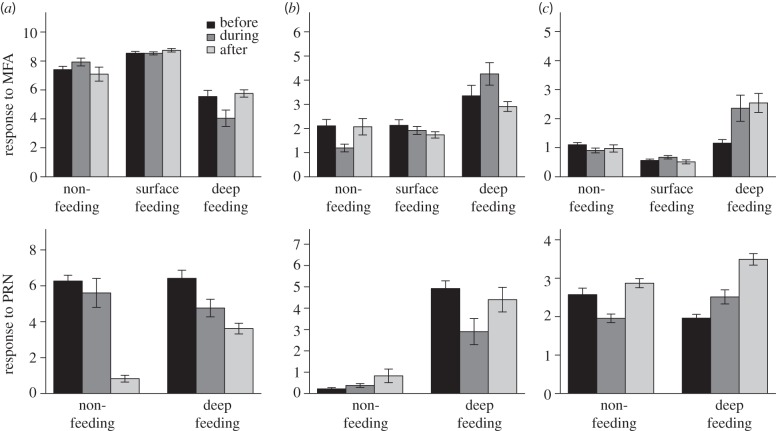
Scaled response metrics. The scaled response is shown for each primary eigenvector within the three parameter groupings ((*a*) diving, (*b*) body orientation and (*c*) horizontal displacement). Each response is shown as a function of CEE treatment status (before, during and after), behavioural state (surface feeding, deep feeding and non-feeding) and sound type (MFA and PRN). Error bars represent 1 s.d. across individuals.

The CEEs were performed on 17 blue whales that were categorized into deep feeding (MFA, *n* = 5; PRN, *n* = 4), shallow feeding (MFA, *n* = 3) and non-feeding (MFA, *n* = 4; PRN, *n* = 1) behavioural states. Our multivariate analyses suggest that several aspects of blue whale diving behaviour (diving, orientation and horizontal displacement metrics) were significantly affected by the exposure to mid-frequency sound (table 2; see also the electronic supplementary material). The responses varied across individuals and were strongly affected by the whale's behavioural state, with surface feeding animals typically showing no change in behaviour (figure 1*a*). By contrast, deep feeding and non-feeding whales were particularly affected, where responses ranged from termination of deep foraging dives (figure 1*b*) to prolonged mid-water dives (figure 1*c*). Responses also varied according to sound type (figure 2 and table 2). For example, blue whales in deep feeding modes exhibited a similar response in diving behaviour and horizontal displacement, but a fundamentally different response was observed with respect to body orientation (figure 2). However, this orientation response in deep feeding whales was transient as behaviour returned to baseline following exposure to both MFA and PRN. Nevertheless, we observed responses that did not return to baseline conditions, at least in the time frame defined by our CEE, for certain combinations of behavioural state and sound type. The overall variability observed here supports previous work demonstrating the complexity of behavioural responses to acoustic signals and its dependence on contextual and sound exposure variables [26].

**Figure 3.**
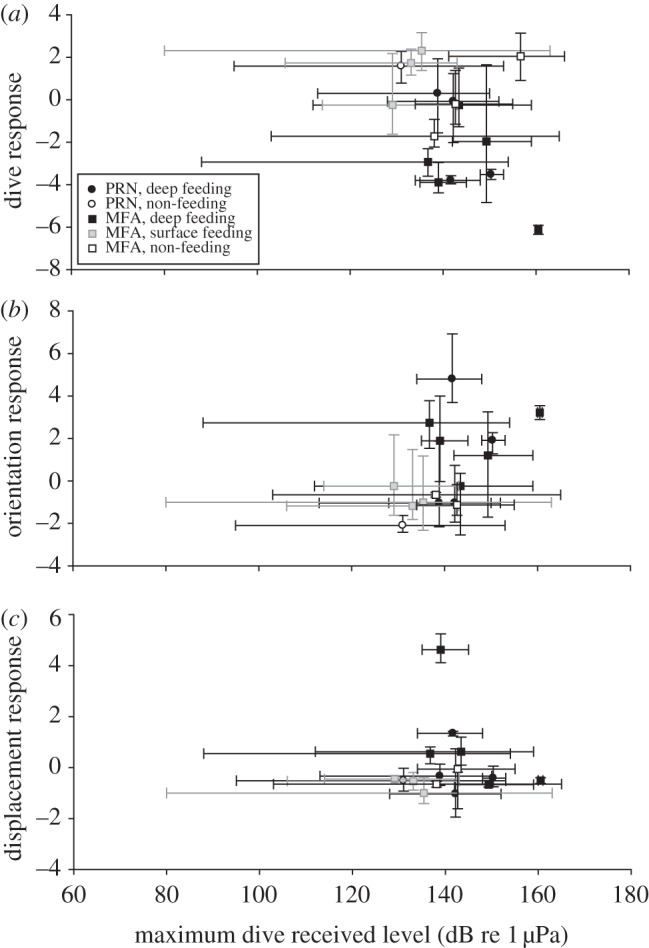
Relationship between maximum received levels on behavioural response. The mean values for the maximum received level measured during a dive are shown as a function of the axis 1 response for (a) diving, (*b*) body orientation and (*c*) horizontal displacement. Each symbol represents values for an individual whale and the error bars indicate the complete range of values from minimum to maximum.

At broad spatial and temporal scales, these context-dependent behavioural responses may be interpreted as brief avoidance responses, but only in particular behavioural states (figure 1). The effects of sound exposure were transient under certain conditions, namely behavioural state and sound type, in that behaviour often returned to pre-exposure conditions after playback ended (figures 1*b,c* and 2). The lack of discernible responses in some surface feeding individuals (figure 1*a*), especially in comparison with deep feeding and non-feeding behavioural modes (figure 1*b,c*), suggest that a combination of behavioural state and received sound level may influence behavioural response. We speculate that surface feeding does not incur sunstantial diving costs and thus blue whales in this behavioural state exhibit increased lunge feeding rates [36] and higher energetic efficiency [32]. The advantages may increase individual motivation to continue exploiting surface krill patches, so blue whale responsiveness to sound in these conditions may be decreased. Although we did find a significant effect of maximum dive received level (maxRL) on the dive response (figure 3*a*), neither body orientation nor horizontal displacement were influenced by maxRL (figure 3*b,c*). Whales near the sea surface were exposed, on average, to lower maxRL on each dive, perhaps resulting from the Lloyd's mirror effect that reduced sonar levels at shallower depths [37]. However, the maximum received sound level experienced over the entire 30 min sound exposure period, in contrast to the maxRL within a given dive, was largely independent of dive depth. These data suggest that the variation in behavioural response is probably influenced by a complex interaction between behavioural state, environmental context and individual differences that may be related to prior exposure to MFA.

These observed effects of mid-frequency sound exposure could have major ramifications for blue whale foraging energetics. For example, the CEE in figure 1*b* shows a blue whale terminating a foraging bout at the onset of sound exposure, followed by directed travel away from the sound source. Because blue whales rely on large aggregations of dense krill to sustain their extreme body size, they continuously dive and feed throughout the day when high-density prey patches are present [38]. Therefore, this type of behavioural response that involves cessation of feeding clearly results in reduced foraging efficiency. Using previously established methods [32], baseline behaviour of this individual prior to playback, and a conservative estimate for krill density, we calculated a feeding rate of 19 kg of krill per minute prior to sound exposure. After the onset of sound exposure, the animal stopped foraging for a total of 62 min, resulting in a loss of over one metric ton of krill during this behavioural response (see the electronic supplementary materials for details). The energy content of this loss is commensurate with the animal's daily basal metabolic demands [39] and thus will predictably decrease the overall efficiency of foraging.

For active sonar operations occurring near blue whale feeding areas, and if there is lack of habituation, repeated exposures could negatively impact individual feeding performance, body condition and ultimately fitness and potentially population health. Although we used MFA signals with temporal and spectral characteristics intended to simulate tactical military systems, operational sonar systems are significantly more intense, mobile, often used with other active sources, and typically used for longer durations. These contextual differences suggest that the effects of real sonar systems could extend for longer and over large geographical regions. Therefore, our results suggest that frequent exposures to mid-frequency anthropogenic sounds may pose significant risks to the recovery rates of endangered blue whale populations, which unlike other baleen whale populations (i.e. humpback, grey and fin whales), have not shown signs of recovery off the western coast of North America in the last 20 years [40].

Like many human activities, MFA sonars represent relatively novel stimuli to cetacean sensory systems that evolved under conditions which were different from present-day environments. Although it is difficult to understand how cetaceans interpret these anthropogenic sounds, previous researchers have invoked the predator evasion hypothesis given the frequency overlap of killer whale S-calls with military sonar signals [41]. Mammal-eating killer whales (*Orcinus orca*) are the only known natural predator of baleen whales [42], and the effects of predation represent a major driving force in the evolution of behaviour [43]. When killer whales attack, *Balaenoptera* whales exhibit a ‘flight’ escape response that is distinct from the ‘stay and fight’ response of *Megaptera* and Balaenidae [42]. The behavioural responses observed here were not comparable in duration to those reported during killer whale attacks on blue whales [42], however, the maximum speed measured in one CEE (figure 1*c*) was similar to previously observed flight speeds. Therefore, it appears that most responses may represent a generalized avoidance response of a perceived threat, rather than a stereotyped flight response. These responses could be influenced by prior exposure to real MFA sonar exercises which are relatively common in these areas off the southern California coast.

Our results provide, to our knowledge, the first experimental demonstration that individual baleen whales, specifically blue whales, respond to simulated mid-frequency sonar. We emphasize that elicitation of the response is complex, dependent on a suite of contextual (e.g. behavioural state) and sound exposure factors (e.g. maximum received level), and typically involves temporary avoidance responses that appear to abate quickly after sound exposure. Based on this evidence, we reject the hypothesis that baleen whales are not affected by military mid-frequency sonar, and in some cases they react at quite low-received levels (figure 1); given their endangered status, blue whales should thus be carefully considered in environmental assessments. Furthermore, the responses we documented were in a geographical region with a high level of naval activity and where mid-frequency sonar use is common, raising the potential for more dramatic responses in other areas if blue whales in this study have habituated. Since some of the most pronounced responses occurred near the onset of exposure but other, higher level exposures provoked no response, the data suggest that the use of received level alone in predicting responses may be problematic and that a more complex dose–response function that considers behavioural contexts will be more appropriate. Management decisions regarding baleen whales and military sonar should consider the likely contexts of exposure and the foraging ecology of animals in predicting responses and planning operations in order to minimize adverse effects.

## Notes

This project was conduced under the terms of US National Marine Fisheries Service (NMFS) research permit no. 14534 (as well as Channel Islands National Marine Sanctuary (CINMS) permit no. 2010-004 for operations within the boundaries of the CINMS).
